# Reframing the paradigm: stereotactic body radiation therapy as an engineer of the tumor immune microenvironment

**DOI:** 10.3389/fimmu.2026.1754669

**Published:** 2026-03-31

**Authors:** Xuehuan Wang, Jianhua Dong, Shaojun Fang, Yao Zhou, Rui Cao, Xianshuo Cheng, Zhibin Yang

**Affiliations:** 1 Department of Colorectal Surgery, The Third Affiliated Hospital of Kunming Medical University/Yunnan Tumor Hospital, Kunming, Yunnan, China; 2 Department of Anesthesiology, The Third Affiliated Hospital of Kunming Medical University, Kunming, Yunnan, China

**Keywords:** cGAS-STING pathway, immunotherapy, liquid biopsy, stereotactic body radiation therapy, tumor immune microenvironment

## Abstract

**Background:**

Stereotactic Body Radiation Therapy (SBRT) is undergoing a paradigm shift from a purely local ablative tool to a systemic immunomodulatory agent. However, a significant knowledge gap remains in understanding how to precisely “engineer” the tumor immune microenvironment (TME) to overcome resistance to immune checkpoint inhibitors (ICIs). Current approaches often rely on empirical combinations rather than biologically guided strategies.

**Methods:**

We conducted a comprehensive narrative review of literature published up to January 2026 using PubMed and Web of Science databases. Keywords included “SBRT,” “immunotherapy,” “cGAS-STING,” “abscopal effect,” and “tumor microenvironment.” We specifically synthesized evidence comparing the immunobiological impacts of ablative versus immunogenic doses (e.g., the TREX1-cGAS-STING axis) and analyzed organ-specific immune tolerance mechanisms.

**Results:**

Evidence suggests a “dose-dependent immunomodulatory window,” where moderate hypofractionation (e.g., 8 Gy x 3) optimally induces type I interferons via the cGAS-STING pathway, whereas single high doses (>12-18 Gy) may dampen immunity through TREX1 induction. Beyond direct cytotoxicity, SBRT remodels the TME by depleting regulatory T cells and recruiting effectors, though this is often counterbalanced by a biphasic influx of MDSCs. Clinical translation faces challenges such as organ-specific immune tolerance, necessitating tailored triplet therapies.

**Conclusion:**

Future success lies in a precision medicine approach: moving beyond generic combinations to “organ-specific triplets” (e.g., adding macrophage-targeting agents in liver disease) and implementing adaptive “closed-loop” protocols where real-time liquid biopsy feedback dictates the timing of the next SBRT pulse.

## 1 Introduction

Stereotactic Body Radiation Therapy (SBRT) has historically been defined by its physical precision and ablative potency (1). However, a paradigm shift is underway, redefining SBRT not merely as a local cytoreductive tool, but as a systemic immunomodulatory agent capable of “engineering” the tumor immune microenvironment (TME) (2). Despite the promise of combining SBRT with immune checkpoint inhibitors (ICIs), clinical results have been mixed, suggesting that a “one-size-fits-all” approach to dosing and scheduling is insufficient (3). A significant knowledge gap remains in understanding how to precisely tailor radiation schemes to overcome organ-specific immune tolerance and resistance mechanisms. Current approaches often rely on empirical combinations rather than biologically guided strategies (4).

To ensure a robust synthesis of evidence addressing these gaps, we conducted a comprehensive narrative review of literature published up to January 2026 using PubMed and Web of Science databases. Keywords included “SBRT,” “immunotherapy,” “cGAS-STING,” “abscopal effect,” and “tumor microenvironment.” We specifically synthesized evidence comparing the immunobiological impacts of ablative versus immunogenic doses (e.g., the TREX1-cGAS-STING axis) and analyzed organ-specific immune tolerance mechanisms to propose a new framework for precision radio-immunotherapy.

## 2 The foundational immunobiology of SBRT: the *in situ* vaccine effect

This section establishes the core biological tenet of SBRT’s immunomodulatory power: its ability to convert an irradiated tumor into an immune-activating hub, an effect termed the *in situ* vaccine.

### 2.1 Induction of immunogenic cell death and pro-inflammatory signal release

SBRT, through the delivery of high-dose radiation, induces a specialized form of apoptosis known as immunogenic cell death (ICD) (5). Unlike conventional apoptosis, ICD is accompanied by the release of potent immunostimulatory molecules.

Damage-Associated Molecular Patterns (DAMPs): During SBRT-induced ICD, critical DAMPs are released. These include surface-exposed calreticulin (CALR) and heat-shock proteins (HSPs), which act as “eat-me” signals for antigen-presenting cells (APCs), and extracellularly released High Mobility Group Box 1 (HMGB1) and adenosine triphosphate (ATP) (6–8). These molecules function as danger signals, binding to pattern recognition receptors (PRRs) on APCs to initiate an inflammatory cascade.

Tumor-Associated Antigens (TAAs): SBRT-induced cell death results in the release of a large bolus of TAAs into the TME and circulation (9). This flood of antigens provides the raw material for the immune system to recognize and mount a specific response against the tumor, effectively creating a personalized *in situ* vaccine.

### 2.2 Activating antigen-presenting cells and priming the adaptive immune response

The combination of DAMPs and TAAs provides a powerful stimulus for dendritic cells (DCs)—the most critical APCs for priming T-cell responses (10). SBRT promotes the maturation of DCs and enhances their cross-presentation of tumor antigens in draining lymph nodes, leading to the activation of naive T-cells (11). This process is fundamental to activating a robust, tumor-specific adaptive immune response that links innate immune activation at the tumor site to systemic, durable immunity.

### 2.3 Upregulation of MHC-I and enhanced tumor cell recognition

Surviving tumor cells within the radiation field are also modulated. SBRT has been shown to increase the expression of Major Histocompatibility Complex Class I (MHC-I) molecules on the surface of cancer cells (12). This upregulation makes tumor cells more “visible” to the immune system, enhancing their recognition and subsequent killing by cytotoxic CD8+ T lymphocytes (CTLs) (13). This mechanism is critical for the effector phase of the anti-tumor immune response.

**Figure 1:**
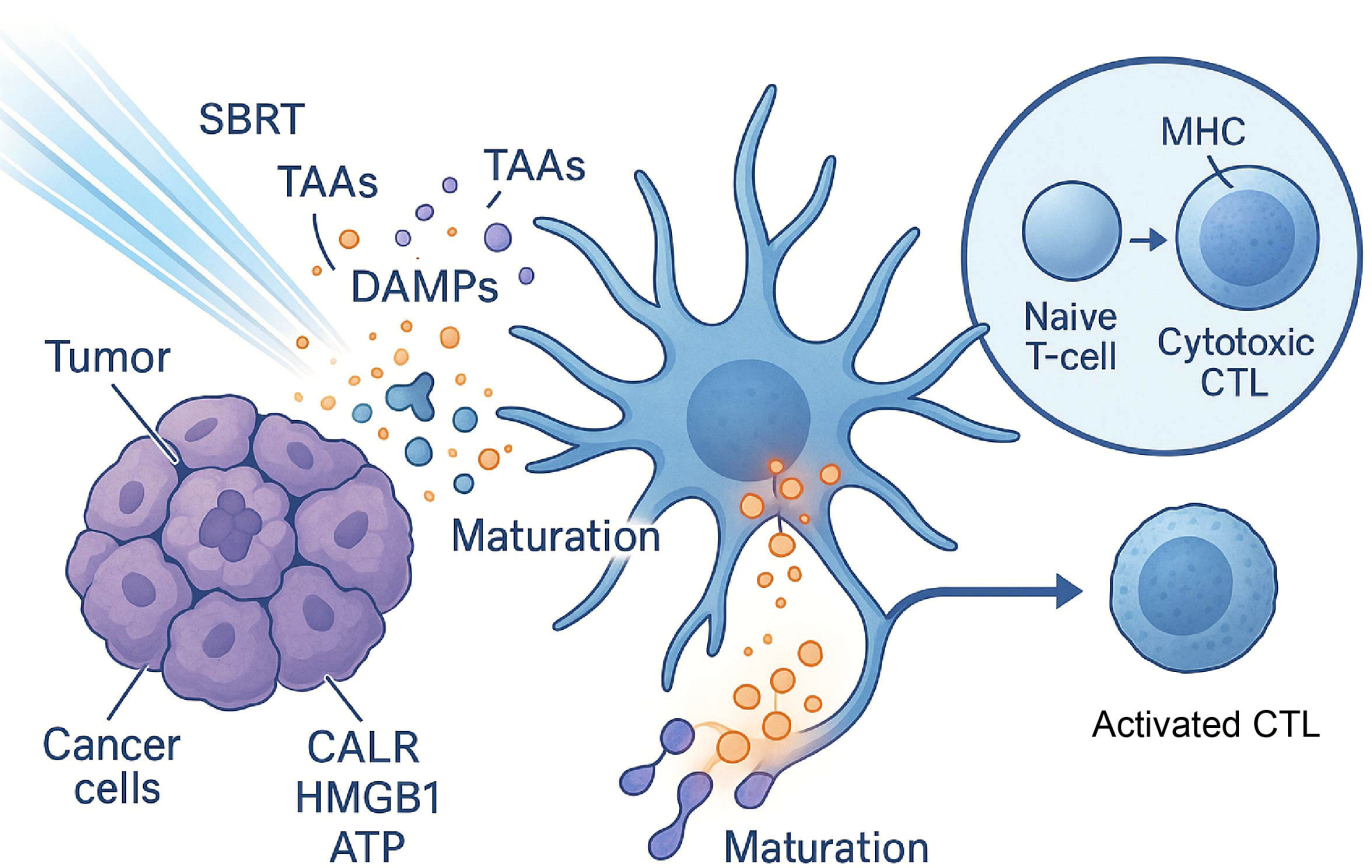
The ‘*in situ* vaccine’ effect of SBRT. Stereotactic Body Radiation Therapy (SBRT) induces immunogenic cell death (ICD) in cancer cells, leading to the release of Damage-Associated Molecular Patterns (DAMPs)—such as surface-exposed calreticulin (CALR), High Mobility Group Box 1 (HMGB1), and ATP—and a bolus of Tumor-Associated Antigens (TAAs). These molecules act as danger signals that promote the maturation of antigen-presenting cells (APCs), such as dendritic cells. Mature APCs then present the TAAs to naive T-cells, priming their activation and differentiation into cytotoxic T lymphocytes (CTLs), which can mediate a systemic anti-tumor immune response.

The *in situ* vaccine effect is not a side effect of SBRT but one of its core mechanisms of action, providing the biological rationale for its combination with immunotherapy. SBRT generates the antigen (the “what to attack”) and the inflammatory signals (the “danger alert”), while ICIs “release the brakes” on the subsequently activated T-cells. This reveals a powerful, multi-step synergy: SBRT creates the ideal upstream conditions (antigen release and DC activation) for ICIs to be effective, and ICIs enhance the downstream effector function of T-cells (14, 15). Without the SBRT-induced immune priming, the population of tumor-specific T-cells for an ICI to act upon may not exist, explaining SBRT’s ability to convert immunologically “cold” tumors into “hot” ones (**Figure 1**).

## 3 The dose-response conundrum: engineering the optimal immunogenic regimen

This section delves into the most critical and nuanced aspect of SBRT as an immune modulator: the profound and often paradoxical effects of dose and fractionation. The central thesis is that maximizing immune stimulation is not synonymous with maximizing radiation dose.

### 3.1 The immunogenic “sweet spot”: why hypofractionated regimens (e.g., 8 Gy x 3) may outperform single ablative doses

Preclinical studies have consistently demonstrated that moderate hypofractionated regimens, such as 8 Gy x 3 fractions, are potent inducers of a Type I Interferon (IFN-I) response, which is a cornerstone of effective anti-tumor immunity (16). In contrast, single, very high doses of radiation can trigger counter-regulatory immunosuppressive mechanisms (17). This introduces the central concept of an “immunomodulatory dose window” that may differ from the dose required for maximal direct cytotoxicity.

### 3.2 The TREX1-cGAS-STING axis: a dose-dependent molecular switch for immunogenicity

The cGAS-STING Pathway: This pathway is a critical sensor of cytosolic DNA. SBRT-induced DNA damage leads to the accumulation of micronuclei and cytosolic DNA fragments, which are detected by cGAS. This triggers the production of cGAMP, activation of STING, and ultimately a robust IFN-I response (18). The IFN-I cascade is essential for recruiting and activating DCs and priming CD8+ T-cells.

TREX1 Induction as a Negative Feedback Loop: Three Prime Repair Exonuclease 1 (TREX1) is a cytosolic nuclease that prevents aberrant activation of the cGAS-STING pathway and autoimmune responses by degrading DNA (19).

The Dose Threshold: Critically, research has identified a dose threshold. Single radiation doses above 12-18 Gy strongly induce the expression of TREX1 in cancer cells (20).

**Figure 2:**
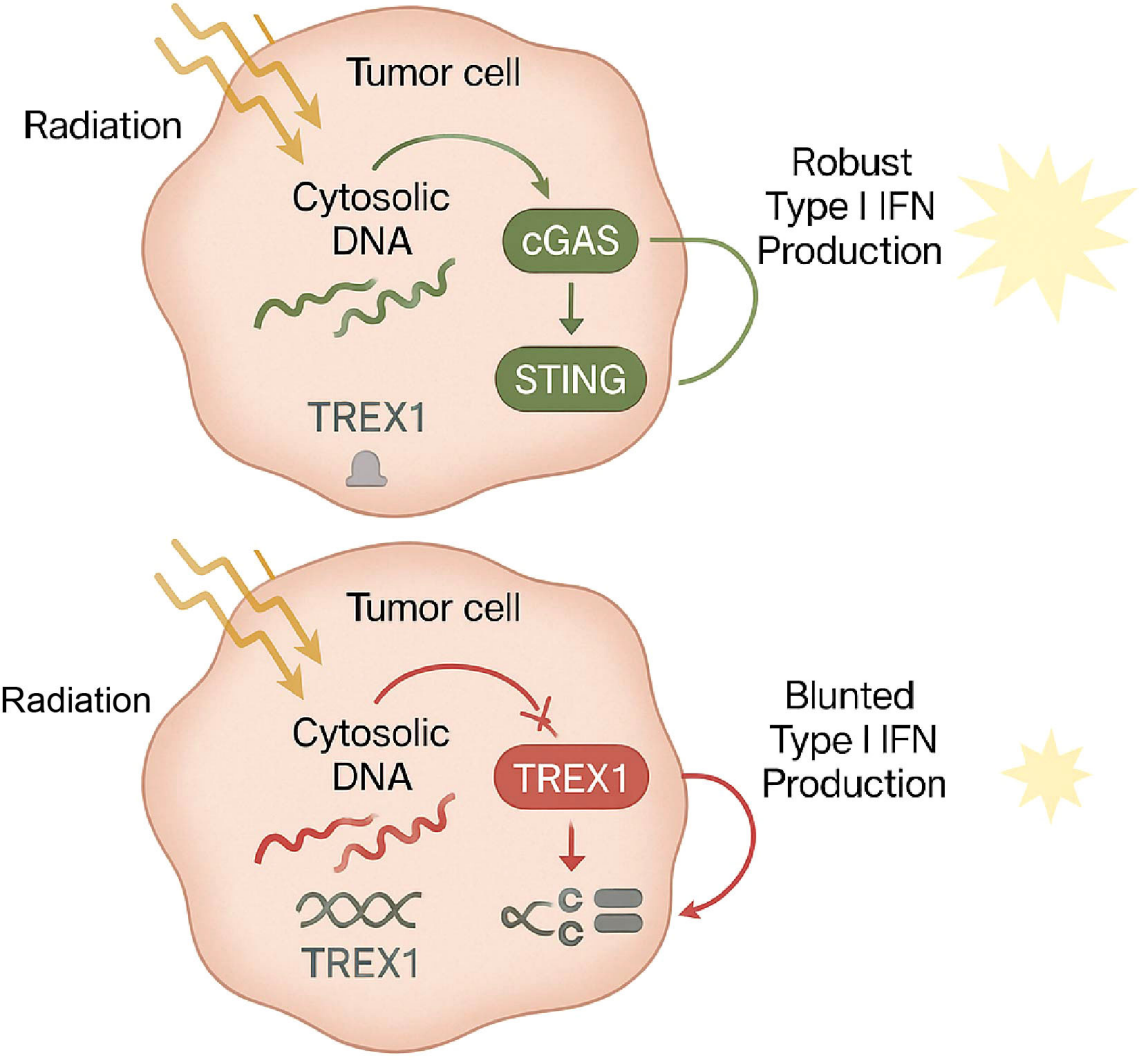
Dose-dependent regulation of the cGAS-STING pathway by TREX1. (Top) Moderate doses of radiation induce the accumulation of cytosolic DNA fragments in tumor cells. This cytosolic DNA is sensed by cGAS, which activates the STING pathway to drive a robust Type I Interferon (IFN-I) response essential for anti-tumor immunity. (Bottom) In contrast, single high doses of radiation (>12-18 Gy) strongly upregulate the exonuclease TREX1. TREX1 degrades cytosolic DNA, thereby preventing cGAS-STING activation and blunting the subsequent IFN-I production, which limits the immunogenicity of the treatment.

The Immunosuppressive Consequence: By degrading the cytosolic DNA generated by high-dose radiation, TREX1 effectively short-circuits the cGAS-STING pathway, blunting the IFN-I response and limiting the immunogenicity of the treatment (21). Conversely, hypofractionated regimens like 8 Gy x 3 have been shown to downregulate TREX1 expression, maximizing cytosolic DNA accumulation and leading to a more potent immune response (**Figure 2**) (16).

### 3.3 Other nucleic acid sensing pathways

It is critical to note that cGAS-STING is not the sole mechanism of radiation-induced sensing. SBRT can also trigger the transcription of endogenous retroviruses (ERVs) and induce mitochondrial DNA leakage (22). These events activate RNA-sensing pathways, specifically RIG-I and MDA5, which operate in parallel with cGAS-STING to amplify Type I interferon signaling. Neglecting these alternative sensing mechanisms may lead to an incomplete understanding of the radiation-induced inflammatory cascade (23).

### 3.4 Beyond ablation: the distinct role of low-dose radiotherapy

It is crucial to distinguish the immunomodulatory mechanisms of ablative SBRT from Low-Dose Radiotherapy (LDRT, typically < 1-2 Gy). While SBRT acts as an *in situ* vaccine causing immunogenic cell death and antigen release, LDRT functions primarily as a TME modulator. LDRT has been shown to repolarize tumor-associated macrophages (TAMs) from an M2-like immunosuppressive phenotype to an M1-like pro-inflammatory state and improve T-cell infiltration by normalizing aberrant tumor vasculature (24). Therefore, a novel “RadScopal” strategy combining high-dose SBRT to the primary tumor (for antigen release) with LDRT to metastatic sites (to remodel the stroma) represents a promising avenue for overcoming systemic resistance. It is important to note that while this combination strategy is currently supported predominantly by robust preclinical mechanistic inference, emerging early-phase clinical evidence has begun to demonstrate LDRT’s ability to reverse tumor immune desertification and overcome resistance to immunotherapy in patients (24). Ongoing and future prospective clinical trials are necessary to fully establish the clinical efficacy and safety of this RadScopal approach.

### 3.5 The complexity of the “immunogenic sweet spot”

While the “8 Gy x 3” regimen is highlighted as a robust inducer of IFN-I, it is important to acknowledge that this is not a universal rule. The optimal immunogenic dose is highly context-dependent, varying by tumor histology, intrinsic DNA repair capabilities (e.g., BRCA mutation status), and the baseline immune landscape. For instance, melanoma models may require different fractionation schemes compared to carcinomas to achieve maximal cGAS-STING activation (25). Thus, the “sweet spot” should be viewed as a biologically defined window rather than a rigid prescription.

### 3.6 Ablative doses and vascular destruction: an indirect pro-inflammatory and cytotoxic mechanism

Beyond direct DNA damage to tumor cells, high-dose SBRT (>8-10 Gy per fraction) has profound effects on the tumor vasculature (26).

Ceramide-Mediated Endothelial Apoptosis: High radiation doses rapidly induce ceramide-mediated apoptosis in tumor endothelial cells, a distinct mechanism from the slower mitotic death observed in tumor cells (27).

Vascular Occlusion and Ischemic Cell Death: This endothelial cell death leads to severe vascular damage, occlusion, and a sharp reduction in tumor perfusion (28). This creates a hypoxic and nutrient-deprived microenvironment, triggering widespread indirect tumor cell death. This vascular mechanism is considered a key contributor to the high efficacy of SBRT, which cannot be fully explained by direct cell-kill models alone (29).

A fundamental tension exists between the two primary goals of SBRT in an immuno-oncology context: direct tumor ablation and immune priming. The very high doses (>12-18 Gy) required for maximal ablation may be suboptimal for immune priming due to TREX1 induction (16). Conversely, the moderate hypofractionated doses that appear most immunogenic may be less ablative. This paradox presents a clinical dilemma, where the choice of dose regimen involves a trade-off between local control and systemic immune potential. Traditional radiobiological concepts like the Biologically Effective Dose (BED), which model direct cell kill, are insufficient to optimize SBRT-immunotherapy combinations (30). A new paradigm, perhaps termed an “Immunologically Effective Dose” (IED), is required. This shifts the therapeutic goal from simply delivering the highest tolerated dose to delivering a dose that is biologically engineered to produce a desired immune phenotype (e.g., high IFN-I, low TREX1). This has profound implications for clinical trial design. As summarized in **Table 1**, future trials should compare not just different total doses, but different fractionation schemes explicitly designed to test specific immunological hypotheses (16, 31).

**Table 1:** The paradigm shift in clinical trial design for SBRT combined with immunotherapy.

Comparison dimension	Traditional trial concept (based on BED)	Future trial concept (based on IED)
Core Therapeutic Goal	Maximizing direct tumor cell kill	Engineering a specific immune phenotype (high IFN-I, low TREX1)
Dose Testing Strategy	Comparing efficacy across different total doses	Comparing specific fractionation schemes explicitly designed to test specific immunological hypotheses
Key Evaluation Metrics	Traditional local tumor control rates	Cytosolic DNA accumulation, ratio of IFN-I to TREX1 expression, and spatial sparing of draining lymph nodes (DLNs)

## 4 Remodeling the cellular landscape of the TME

This section transitions from molecular mechanisms to the cellular level, examining how SBRT alters the balance of power between pro- and anti-tumor immune cell populations.

### 4.1 Mobilizing the attack: enhancing effector T-cell and NK cell function

A primary goal of SBRT is to convert a T-cell-poor, “cold” TME into a T-cell-infiltrated, “hot” TME (14). Through chemokine release and cGAS-STING activation, SBRT promotes the infiltration of cytotoxic CD8+ T-cells, the primary effectors of anti-tumor immunity (32). Studies show SBRT can increase the diversity of the T-cell receptor (TCR) repertoire, suggesting the priming of new T-cell clones against a broader array of tumor antigens (33). Furthermore, SBRT also impacts innate immunity, with some evidence suggesting it promotes the infiltration and activation of Natural Killer (NK) cells, providing a rapid, non-specific anti-tumor response (34).

### 4.2 Dismantling the defenses: the complex dynamics of Tregs and MDSCs

Regulatory T-Cells (Tregs): Marked by FOXP3, Tregs are a major immunosuppressive population that dampens anti-tumor responses. Studies show that SBRT can decrease levels of immunosuppressive markers like FOXP3, indicating a depletion or functional inhibition of Tregs within the TME (35). This “disarming” of the TME is critical for allowing effector T-cells to function.

Myeloid-Derived Suppressor Cells (MDSCs): The effect of SBRT on MDSCs is more complex and seemingly contradictory.

Suppressive Effects: Some preclinical evidence suggests SBRT can suppress MDSCs. One study found SBRT upregulates miR-21, which in turn inhibits MDSC differentiation and promotes their apoptosis (36). Other clinical observations show decreased MDSC counts following SBRT using higher single-fraction doses (37).

Recruitment Effects: However, a substantial body of evidence indicates that the inflammatory environment created by radiotherapy can actively recruit MDSCs to the tumor site. Radiation-induced cGAS-STING activation and cytokine/chemokine release (e.g., CCL2, CCL5) are potent signals for MDSC mobilization from the bone marrow (38). This can create a negative feedback loop where the pro-inflammatory effects of SBRT are counteracted by a new influx of immunosuppressive cells.

Crosstalk: MDSCs and Tregs engage in bidirectional crosstalk, where each cell type promotes the function and accumulation of the other, forming a potent immunosuppressive axis that SBRT must overcome (39).

**Figure 3:**
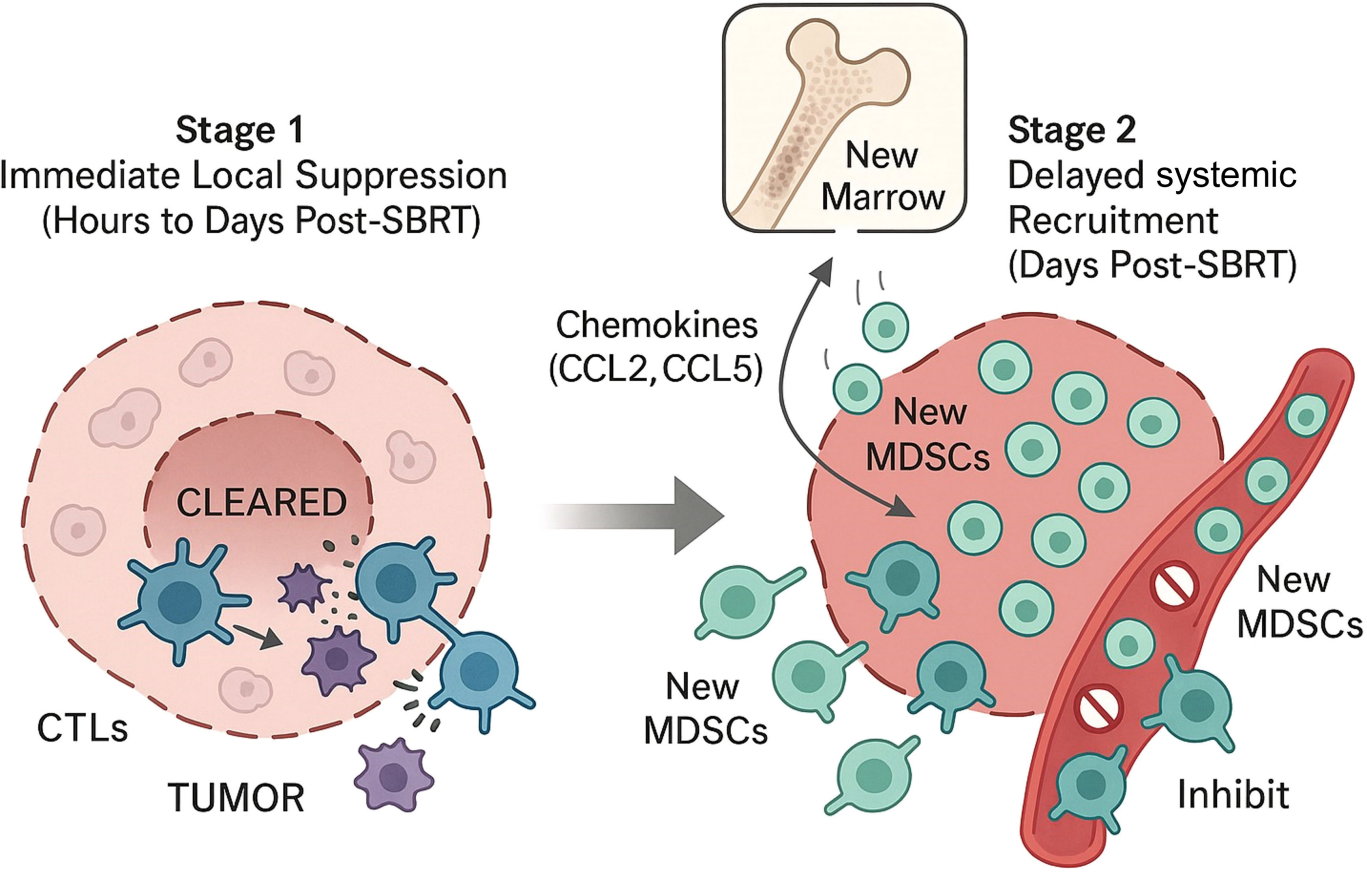
The biphasic response model of Myeloid-Derived Suppressor Cells (MDSCs) following SBRT. The effect of SBRT on MDSCs can be conceptualized in two distinct stages. Stage 1: Immediate Local Suppression (Hours to Days Post-SBRT) involves the direct cytotoxic effect of radiation on MDSCs and tumor cells within the treatment field, leading to their clearance. Stage 2: Delayed Systemic Recruitment (Days Post-SBRT) is characterized by the release of inflammatory chemokines (e.g., CCL2, CCL5) from the irradiated tumor, which mobilizes new MDSCs from the bone marrow and recruits them back to the tumor microenvironment, where they exert potent immunosuppressive functions.

The apparently contradictory data on MDSCs are not a flaw in the research but point to a complex, biphasic biological process. It is likely that SBRT first exerts a direct, local suppressive/cytotoxic effect on MDSCs within the treatment field, followed by a secondary, systemic recruitment of new MDSCs driven by the inflammatory response (36). This reveals a dynamic therapeutic window of opportunity: immediately following SBRT, the TME may be transiently cleared of MDSCs, but this is followed by a wave of new suppressive cells (36). This understanding of a biphasic MDSC response provides a strong biological rationale for optimizing the timing of combination therapies. Rather than co-administering MDSC-targeting agents (e.g., CXCR2 or CCR2 inhibitors) concurrently with SBRT, a sequential administration several days after SBRT completion, designed specifically to intercept the wave of newly recruited MDSCs, may be a more effective strategy (40). This approach turns a “contradiction” in the data into a testable and clinically meaningful therapeutic hypothesis (**Figure 3**).

### 4.3 Beyond T-cells: neutrophils, macrophages, and cancer-associated fibroblasts

SBRT-induced remodeling extends to the broader stromal compartment. Neutrophils: Radiation can trigger the release of Neutrophil Extracellular Traps (NETs), which may paradoxically shield tumor cells from immune attack and promote metastasis (41). This highlights a potential target for combination therapy using NET inhibitors. Cancer-Associated Fibroblasts (CAFs): SBRT can stiffen the extracellular matrix via CAFs, potentially hindering T-cell migration. However, specific doses may also render CAFs pro-inflammatory, suggesting a complex plasticity that requires further investigation (42). Spatial Distribution: Importantly, effective SBRT does not just increase the number of immune cells but alters their spatial distribution, facilitating the migration of CD8+ T-cells from the invasive margin into the tumor core, overcoming the “immune-excluded” phenotype (43).

Macrophages are a dominant component of the stromal landscape. Radiation exerts a dual effect on TAMs depending on the dose (44). While low doses (LDRT) can promote the repolarization of pro-tumor M2 macrophages toward an anti-tumor M1 phenotype, high ablative doses may trigger the recruitment of distinctive monocytes (CCR2+) that rapidly differentiate into immunosuppressive macrophages to repair tissue damage (45). This necessitates strategies that block macrophage recruitment (e.g., CCR2 inhibitors) or reprogramming (e.g., PI3Kγ inhibitors) when high-dose SBRT is employed.

### 4.4 The role of draining lymph nodes: to irradiate or to spare?

A critical consideration in SBRT planning is the management of Draining Lymph Nodes (DLNs). The DLNs are the primary sites for T-cell priming by dendritic cells carrying SBRT-released antigens. Elective nodal irradiation (ENI) utilizing high doses may inadvertently sterilize this critical immune hub, blunting the systemic abscopal response. Recent preclinical data suggest that sparing the DLNs—or treating them with strictly low-dose modulation—preserves the specific stem-like T-cell populations necessary for a durable response to immunotherapy (46). This advocates for a more organ-sparing approach to volume delineation in the era of immuno-radiotherapy.

At present, there is no universally accepted clinical dosimetric definition of DLN “sparing,” and numeric thresholds (e.g., mean dose < 2 Gy or V5 < 10%) should be considered hypothesis-generating rather than evidence-based constraints. Nevertheless, converging preclinical and early translational evidence indicates that concomitant irradiation of tumor-draining lymph nodes can disrupt immune priming and attenuate systemic responses, whereas strategies that spare DLNs or temporally delay DLN irradiation may preserve radio-immunotherapy efficacy (47). Therefore, a pragmatic near-term approach is to treat DLNs as immune organs-at-risk: (i) prospectively define the most likely draining basins using anatomical drainage patterns and/or sentinel-node mapping when feasible; (ii) minimize low-dose “bath” to these regions during plan optimization (reduce mean dose and V5–V10 whenever oncologically safe); and (iii) record DLN dose–volume metrics in clinical trials as candidate immune constraints.

Key directions for future research include standardized DLN contouring atlases, prospective correlation of DLN dose–volume with T-cell priming/IFN–ISG readouts, and clinical testing of DLN-sparing or delayed-DLN strategies in settings where nodal control can be maintained without compromising oncologic outcomes (48, 49).

## 5 Clinical translation: synergy and challenges of the SBRT-immunotherapy combination

This section bridges preclinical science with clinical reality, evaluating the evidence for SBRT-ICI synergy, the major obstacles to its successful application, and data from key clinical trials.

### 5.1 Overcoming resistance: converting immunologically “cold” tumors to “hot”

Many tumors are inherently resistant to ICIs due to a lack of pre-existing T-cell infiltration (a “cold” TME) or low PD-L1 expression (50). SBRT, as an immune primer, can generate the inflammation and T-cell infiltration necessary for ICIs to work (51). The PEMBRO-RT trial, for instance, suggested that the benefit of adding SBRT to pembrolizumab was most pronounced in patients with low baseline PD-L1 expression.

The phase II PEMBRO-RT trial in NSCLC randomized patients to pembrolizumab alone or combined with SBRT (8 Gy x 3). Although the study narrowly missed its prespecified primary endpoint, the experimental arm showed a consistent trend toward improvement in Objective Response Rate (ORR: 36% vs. 18%, P = 0.07) and Median Progression-Free Survival (mPFS: 6.6 vs. 1.9 months, HR 0.71, P = 0.19) (52). Subgroup analysis notably revealed that the benefit was driven almost entirely by patients with PD-L1 negative tumors. Similarly, the MD Anderson randomized phase II trial combining SBRT (50 Gy/4 fx) with pembrolizumab reported an ORR of 38% vs. 20% and a significantly improved mPFS (6.4 vs. 1.8 months, P = 0.04), providing further evidence of systemic efficacy outside the radiation field (53). However, not all trials have been positive, underscoring the need to refine patient selection based on biomarkers rather than anatomy alone.

### 5.2 The organ-specific battlefield: deconstructing the unique immunosuppressive challenge of the liver

Clinical data consistently show that patients with liver metastases respond significantly worse to ICIs, with lower objective response rates (ORR) and progression-free survival (PFS) compared to patients with lung or lymph node metastases (54).

The Liver’s Immune Tolerant Microenvironment: The liver is not just immunologically “cold”; it is an actively immune tolerant organ, evolutionarily designed to prevent excessive immune reactions to antigens absorbed from the gut (55). This pre-existing state of immunosuppression poses a formidable challenge, as SBRT-induced inflammation may trigger powerful homeostatic feedback mechanisms that reinforce, rather than break, this tolerance. (**Table 2**) (56).

**Table 2:** Comparison of immune microenvironments and targeted strategies in lung versus liver metastases.

Features and strategies	Lung cancer immune microenvironment	Liver immune microenvironment
Immune Status Characteristics	Adaptive immune resistance (predominantly PD-L1 expression)	Innate immune tolerance (evolutionarily designed to prevent excessive immune reactions to antigens absorbed from the gut)
Core Therapeutic Challenge	Re-starting a stalled T-cell response (mechanism: releasing the brakes)	Overcoming innate tolerance to permit T-cell priming (mechanism: building the car)
Recommended Combination Strategy	Doublet therapy (SBRT and ICI)	Triplet therapy (SBRT, ICI, and agents targeting myeloid or stromal cells)

The profound differences in immune architecture between organs like the lung and liver mean that a “one-size-fits-all” approach to SBRT-immunotherapy is destined to fail (57). Therapeutic strategies must be tailored to the specific organ microenvironment. For example, the TME in non-small cell lung cancer (NSCLC) often exhibits adaptive immune resistance (e.g., PD-L1 expression), where SBRT+ICI works by re-starting a stalled T-cell response (58). The liver TME, however, is characterized by innate immune tolerance driven by myeloid and stromal cells, where a T-cell response to re-start may not exist in the first place (59). Thus, in the lung, the problem is to “release the brakes” on T-cells; in the liver, the problem is to “build the car” (i.e., overcome innate tolerance to permit T-cell priming) (60). To successfully treat liver metastases, a third agent specifically targeting the liver’s unique biology—such as targeting myeloid cells (e.g., CSF1R inhibitors), stromal cells (e.g., FAK inhibitors), or specific cytokine pathways (e.g., TGF-β inhibitors)—may be required. This suggests that future clinical trials should move beyond simple SBRT+ICI combinations toward rationally designed, organ-specific triplet therapies. To achieve this, identifying precise immune and hypoxic biomarkers through baseline bioinformatic profiling—as demonstrated in recent colorectal cancer models (61, 62) —is crucial to stratify patients who might benefit most from combining SBRT with ICI and stromal-targeting agents.

### 5.3 Review of landmark and ongoing clinical trials

A systematic review of pivotal trials reveals the evolving landscape of SBRT-ICI combinations, as detailed in **Table 3**. Early phase studies, such as those in metastatic NSCLC and melanoma, primarily focused on safety and established that concurrent SBRT with PD-1/PD-L1 blockade is well-tolerated without synergistic toxicity (63). However, efficacy data have been heterogeneous. While the PEMBRO-RT trial suggested a benefit in PD-L1 negative patients, larger randomized trials and ongoing phase III studies (e.g., NRG-LU002) continue to investigate the optimal sequencing and patient selection to validate these signals (52). Notably, recent trials are increasingly incorporating “triplet” strategies—combining SBRT, ICI, and a targeted agent (e.g., GM-CSF or cytokine modulators)—to overcome the limitations of doublet therapy (64). The discordant results across trials highlight that distinct tumor histologies and anatomical sites (e.g., lung vs. liver) require distinct radio-immunotherapy prescriptions, moving away from a universal protocol (65).

**Table 3:** Summary of landmark and representative clinical trials of SBRT combined with immunotherapy.

Trial phase and name	Target cancer type	Treatment regimen	Key findings and research focus
Early-Phase Studies	Metastatic NSCLC and melanoma	Concurrent SBRT with PD-1/PD-L1 blockade	Established that concurrent SBRT with PD-1/PD-L1 blockade is well-tolerated without synergistic toxicity.
PEMBRO-RT (Phase II)	NSCLC	Pembrolizumab alone or combined with SBRT (8 Gy x 3)	Demonstrated a trend toward improvement in Objective Response Rate and Median Progression-Free Survival; the benefit was driven almost entirely by patients with PD-L1 negative tumors.
MD Anderson (Phase II Randomized)	Metastatic NSCLC	Pembrolizumab with or without SBRT (50 Gy/4 fx)	Reported significantly improved ORR and mPFS, providing further evidence of systemic efficacy outside the radiation field.
Phase III Trials (NRG-LU002)	NSCLC	SBRT and Immunotherapy	Investigating the optimal sequencing and patient selection to validate early phase signals.
Recent “Triplet Therapy” Trials	Multiple solid tumors	SBRT, ICI, and a targeted agent (GM-CSF or cytokine modulators)	Incorporating triplet strategies to overcome the limitations of doublet therapy.

## 6 New frontiers: advanced strategies and future outlook

**Figure 4:**
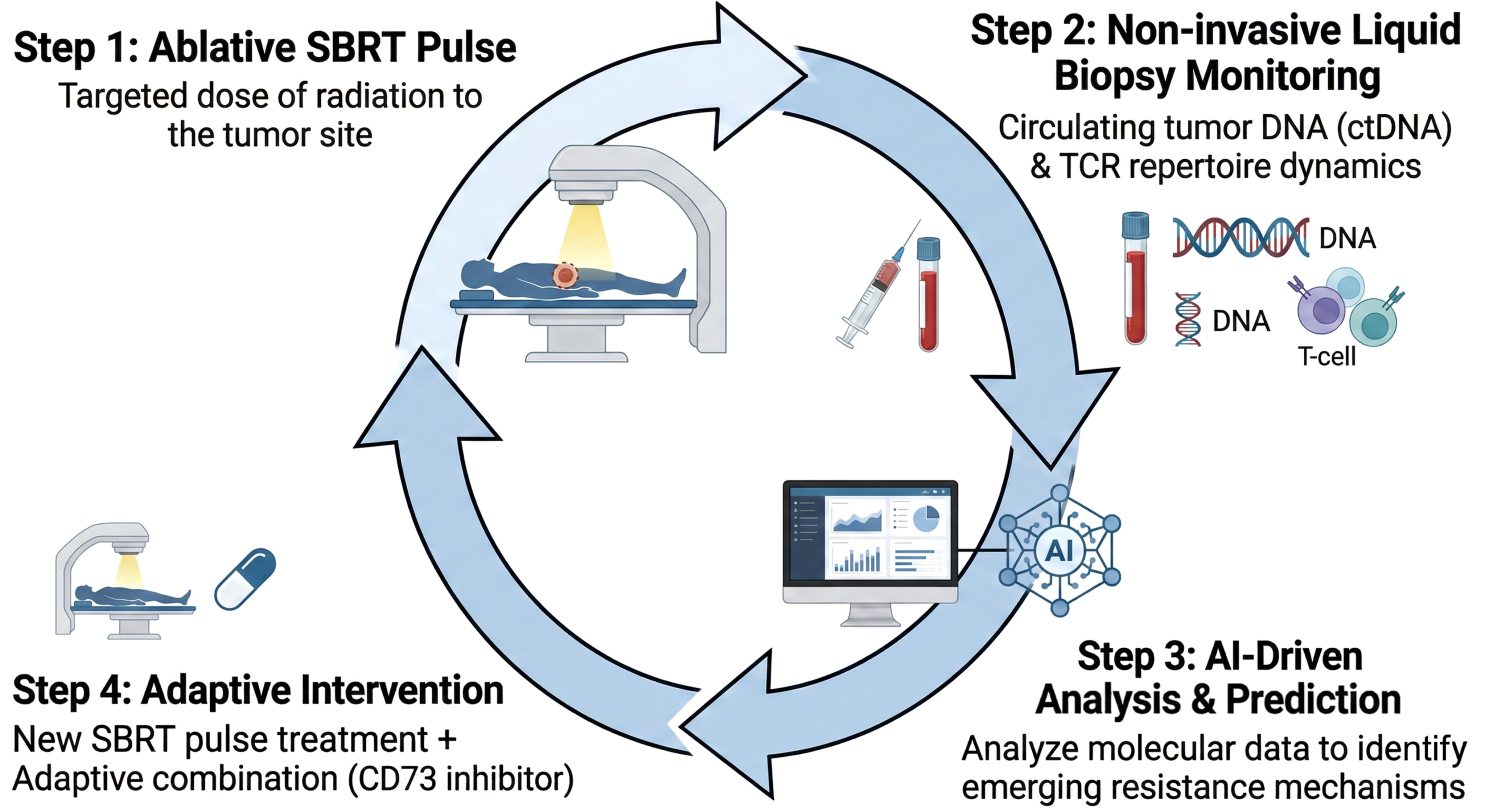
A proposed closed-loop paradigm for personalized adaptive radioimmunotherapy. This model illustrates a future strategy for cancer treatment guided by real-time biological feedback. Step 1: An ablative pulse of SBRT is delivered to the tumor. Step 2: Treatment response is monitored non-invasively using liquid biopsies to analyze circulating tumor DNA (ctDNA) kinetics and T-cell receptor (TCR) repertoire dynamics. Step 3: AI-driven computational models analyze these data to predict tumor response and identify emerging resistance mechanisms. Step 4: The analysis informs the selection and timing of the next therapeutic intervention, such as an adaptive combination therapy (e.g., adding a CD73 inhibitor) with the subsequent SBRT pulse, creating a personalized and dynamic treatment cycle.

This section explores cutting-edge research and concepts that are pushing the boundaries of SBRT as an immune-engineering tool (**Figure 4**).

### 6.1 Defining the immunologically effective dose

To advance the field, we propose the formal adoption of the “Immunologically Effective Dose” (IED) concept. Unlike the Biologically Effective Dose (BED) which focuses on cell kill, the IED incorporates parameters such as (1): quantification of cytosolic DNA accumulation; (2) ratio of IFN-I to TREX1 expression and (3) spatial sparing of Draining Lymph Nodes (DLNs). Consequently, the concept of IED should be integrated into a broader “Immunologically Effective Radiotherapy” (IER) plan that optimizes both the dose delivered to the tumor and the dose spared from critical immune tissues.

Practical measurement of IED in patients remains non-trivial, but several translational “toolboxes” are now feasible. First, cytosolic DNA accumulation can be approximated in paired tumor biopsies by quantifying radiation-induced micronuclei and cGAS localization using multiplex immunofluorescence or spatial imaging, and by measuring downstream pathway activation through an interferon/ISG transcriptional program (e.g., CXCL10, IFIT1, ISG15) using RNA-based assays. Notably, biopsy-based assessment of radiation-induced cGAS re-localization and IFN/ISG immune signatures has been linked to clinical efficacy in human HNSCC, providing proof-of-principle that these mechanistic readouts can be captured in patients (66). Similarly, in NSCLC treated with pembrolizumab plus radiotherapy, systemic immune activation has been monitored longitudinally through blood immunophenotyping and immune profiling, supporting a clinically implementable framework for immune response tracking (67).

Second, the IFN-I/TREX1 ratio is conceptually attractive but is unlikely to be reliably inferred from plasma alone because TREX1 is primarily intracellular. A more robust approach is to quantify TREX1 at the tumor level (IHC or RNA) and normalize it to a contemporaneous IFN/ISG signature in the same biopsy specimen, while using blood-based IFN/ISG scores as a complementary systemic surrogate. For minimally invasive monitoring, candidate liquid-biopsy surrogates include (i) whole-blood or PBMC IFN/ISG scores, (ii) serum cytokine/chemokine panels enriched for IFN-driven factors (e.g., CXCL10), and (iii) orthogonal response kinetics from ctDNA and TCR repertoire dynamics, which reflect antigen release and clonal expansion even when direct pathway readouts are not obtainable (67).

Third, operationalizing “DLN sparing” for IED requires prospectively defining a pragmatic DLN region (based on anatomical drainage patterns and/or sentinel-node mapping when available) and routinely reporting low-dose metrics (e.g., mean dose, V5–V10) as candidate immune constraints. This concept aligns with the broader movement toward quantifying dose to the immune compartment (e.g., EDIC-style frameworks) and enables prospective correlation between immune-dose metrics and immunologic endpoints (68).

### 6.2 Optimizing the fourth dimension: the critical question of SBRT-immunotherapy timing

The optimal timing of SBRT administration relative to ICI remains a critical and unresolved question (69). Rationale for SBRT First: Administering SBRT first allows the *in situ* vaccine effect to prime a T-cell response, which can then be unleashed by subsequent checkpoint inhibition.

Rationale for Concurrent Therapy: Concurrent administration may capture the peak of inflammation and T-cell infiltration, and a pre-existing T-cell population may be critical for radiation to exert its maximal effect (70).

Clinical Evidence: One retrospective study found significantly worse overall survival in patients who completed immunotherapy prior to SBRT/SRS compared to those receiving it concurrently or after, suggesting that irradiating a tumor in an immune-exhausted state is suboptimal (71).

### 6.3 Personalized adaptive radiotherapy: The PULSAR concept and future scheduling

PULSAR (Personalized Ultrafractionated Stereotactic Adaptive Radiotherapy): This innovative concept abandons standard daily fractionation in favor of delivering ablative doses in “pulses” separated by long time intervals (e.g., 10 days) (72).

Biological Rationale: The long interval is designed to match the kinetics of the adaptive immune response, allowing the T-cell response generated by the first pulse to mature and reach full effect before the next pulse is delivered (72).

Preclinical Evidence: Preclinical models show that PULSAR-style scheduling, when combined with α-PD-L1, achieves superior tumor control and induces immunologic memory compared to traditional daily fractionation (73). This represents a shift in radiotherapy scheduling from being based on radiobiological convenience to being based on immunological timing.

### 6.4 New combinations for intractable cancers: targeting the adenosine and LIF pathways in pancreatic cancer

Pancreatic ductal adenocarcinoma (PDAC) is a classically “cold” tumor, highly resistant to immunotherapy and characterized by a dense, immunosuppressive stroma (74). SBRT plus ICI alone has had limited success (75).

Targeting the Adenosine Pathway: The PDAC TME is rich in extracellular adenosine, a potent immunosuppressive molecule generated by the ectoenzymes CD39 and CD73. Research has shown that SBRT upregulates CD73 on pancreatic cancer cells, creating a resistance mechanism (76). Preclinical studies show that a triple therapy of SBRT + anti-PD-L1 + a CD73 inhibitor significantly improves tumor control and survival in PDAC models, including control of liver metastases (77).

Targeting the LIF Pathway: Leukemia Inhibitory Factor (LIF) is another cytokine implicated in the immunosuppressive TME of PDAC, promoting Treg differentiation and M2 macrophage polarization (78). Preclinical models show that combining chemotherapy with dual LIF and PD-L1 blockade enhances anti-tumor immunity (79). This provides a strong rationale for investigating a triple combination of SBRT + anti-PD-L1 + a LIF inhibitor to comprehensively remodel the PDAC TME.

### 6.5 Expanding the arsenal: SBRT with cellular therapies and oncolytic viruses

The “engineering” capability of SBRT extends beyond ICIs. CAR-T Therapy: Solid tumors often resist CAR-T therapy due to antigen heterogeneity and physical barriers. SBRT can address this by debulking the tumor mass and inducing “antigen spreading,” potentially preventing antigen-negative relapse. Furthermore, SBRT-induced vascular normalization may improve CAR-T cell trafficking into the tumor core (80). However, CAR-T therapy carries risks of cytokine release syndrome (CRS) and neurotoxicity; thus, SBRT sequencing should be designed to minimize overlapping inflammatory peaks around CAR-T infusion, with SBRT most commonly positioned as cytoreductive/bridging therapy prior to infusion and with careful post-infusion toxicity monitoring (81). Oncolytic Viruses (OVs): The combination of SBRT and OVs represents a dual-danger signal strategy. SBRT can enhance viral replication and oncolysis, while the virus amplifies the inflammatory cascade initiated by radiation, creating a potent synergy particularly in immunologically cold environments (82).

### 6.6 Dynamic monitoring with liquid biopsies: guiding therapy with ctDNA and TCR repertoire analysis

Traditional imaging provides a slow assessment of response and can be confounded by phenomena like pseudoprogression (83). Liquid biopsies offer a dynamic, real-time window into treatment response.

T-Cell Receptor (TCR) Repertoire Sequencing: Serial TCR sequencing can track the diversity and clonal expansion of T-cells in the peripheral blood. A positive response to SBRT-immunotherapy is associated with decreased or stable TCR diversity (indicating expansion of dominant anti-tumor clones) and the emergence of new, high-abundance TCR clones (reflecting the *in situ* vaccine effect) (84). Conversely, disease progression is associated with an increase in diversity as these clones contract.

Circulating Tumor DNA (ctDNA): Monitoring ctDNA levels provides a highly sensitive measure of tumor burden. A rapid drop in ctDNA often precedes radiologic response and can help distinguish true progression from pseudoprogression (85). Studies have shown that a transient spike in ctDNA post-SBRT (from cell death) followed by a rapid clearance is a marker of good response (86). Molecular progression detected by ctDNA can precede radiologic progression by weeks or months, providing an early warning of treatment failure (87).

The future strategies discussed in this section—PULSAR, novel molecular combinations, and liquid biopsy monitoring—are not isolated concepts; they converge toward a fully personalized and adaptive treatment paradigm. Liquid biopsies (ctDNA/TCR) provide real-time feedback on a patient’s biological response to therapy (88). This feedback can be used to adjust the therapeutic strategy on the fly. For example, if TCR analysis after a first SBRT pulse shows a failure to expand new clones, a different molecular agent (e.g., a CD73 inhibitor) could be added. The PULSAR framework, with its long inter-fraction intervals, provides the ideal temporal structure to perform this monitoring and adaptation (89). One could measure the immune response after the first pulse and use that data to inform the timing and combination strategy for the second pulse. The ultimate evolution of this trend is a “closed-loop” treatment system. In this paradigm, a patient receives a pulse of SBRT. Days later, a liquid biopsy is performed. The molecular data (ctDNA kinetics, TCR repertoire changes) are fed into a predictive model (perhaps AI-driven, as hinted at in (90)) that determines the optimal timing for the next SBRT pulse and the specific immunotherapy agent that should be paired with it to overcome observed resistance mechanisms. This represents a true TME engineering approach, where the system is actively measured, modeled, and modulated in a continuous, adaptive cycle.

### 6.7 Challenges in clinical implementation

Despite the promise, the clinical utility of ctDNA/TCR monitoring faces substantial hurdles. First, the “shedding dynamics” vary significantly across tumor types; for example, intracranial metastases often shed minimal DNA into plasma due to the blood-brain barrier (91). Second, differentiating a post-SBRT “flare” (a transient spike in ctDNA caused by cell lysis) from true biological progression remains difficult without standardized time-point definitions (92). Finally, the lack of uniform assay sensitivity and high costs currently limit widespread routine adoption. Future protocols must validate specific “molecular response criteria” before these biomarkers can guide treatment changes outside of clinical trials (93).

## 7 Conclusion and strategic recommendations

This review synthesizes the evidence for a new paradigm of SBRT as an immune-engineering modulator. It reiterates that the optimal use of SBRT in this context requires a shift from a “maximum dose” philosophy to one of a “biologically tailored dose.”

### 7.1 Recommendations for clinical practice

Optimize Fractionation: When immune synergy is a primary goal, moderate hypofractionation regimens (e.g., 8 Gy x 3) should be considered to maximize immune stimulation and avoid high-dose-induced TREX1-mediated suppression.

Multidisciplinary Management: For patients with highly immunosuppressive sites like liver metastases, a multidisciplinary approach should be emphasized, considering the addition of a third agent targeting myeloid cells or the stroma to the SBRT and ICI backbone.

Judicious Use of Biomarkers: Dynamic biomarkers like ctDNA can be judiciously used in clinical decision-making to help differentiate pseudoprogression from true progression and provide an early assessment of efficacy.

### 7.2 Directions for future research

Head-to-Head Fractionation Trials: Prospective, randomized trials are urgently needed to directly compare different SBRT fractionation schemes on immunological endpoints (e.g., IFN-I levels, T-cell infiltration), not just traditional tumor control.

Rational Triplet Therapy Trials: In immunologically challenging settings like pancreatic cancer and liver metastases, trials of “triplet therapies” based on clear biological rationales, such as SBRT + ICI + CD73 or LIF inhibitors, should be designed.

Integrating Adaptive Radiotherapy and Dynamic Monitoring: Clinical trial designs that combine adaptive radiotherapy concepts like PULSAR with intensive liquid biopsy monitoring should be pursued to pioneer the next generation of personalized radioimmunotherapy.

The ultimate goal is to move from empirically combining SBRT and immunotherapy to rationally engineering a desired, systemic anti-tumor immune response. Currently, based on the “Immunologically Effective Dose” (IED) and organ-specific paradigms discussed in this review, our center is initiating a prospective clinical study to validate these combinatorial strategies in metastatic colorectal cancer patients.

## References

[1] Timmerman RD Herman J Cho LC . Emergence of stereotactic body radiation therapy and its impact on current and future clinical practice. J Clin Oncol. (2014) 32:2847–54. doi: 10.1200/jco.2014.55.4675. PMID: 25113761 PMC4152712

[2] Nelson BE Adashek JJ Lin SH Subbiah V . The abscopal effect in patients with cancer receiving immunotherapy. Med. (2023) 4:233–44. doi: 10.1016/j.medj.2023.02.003. PMID: 36893753 PMC10116408

[3] Lynch C Korpics MC Katipally RR Wu T Bestvina CM Pitroda S . Combined stereotactic body radiation therapy and immune checkpoint inhibition for liver metastases: Safety and outcomes in a pooled analysis of 3 phase 1 trials. Int J Radiat Oncol Biol Phys. (2024) 118:1519–30. doi: 10.1016/j.ijrobp.2024.01.002. PMID: 38199382

[4] Demaria S Guha C Schoenfeld J Morris Z Monjazeb A Sikora A . Radiation dose and fraction in immunotherapy: one-size regimen does not fit all settings, so how does one choose? J Immunother Cancer. (2021) 9:002038. doi: 10.1136/jitc-2020-002038. PMID: 33827904 PMC8031689

[5] Xiao Y Zhuang H . Effect of stereotactic radiotherapy on immune microenvironment of lung cancer. Front Immunol. (2022) 13:1025872. doi: 10.3389/fimmu.2022.1025872. PMID: 36211382 PMC9540518

[6] Passelli K Repáraz D Kinj R Herrera FG . Strategies for overcoming tumour resistance to immunotherapy: harnessing the power of radiation therapy. Br J Radiol. (2024) 97:1378–90. doi: 10.1093/bjr/tqae100. PMID: 38833685 PMC11256940

[7] Vaes RDW Hendriks LEL Vooijs M De Ruysscher D . Biomarkers of radiotherapy-induced immunogenic cell death. Cells. (2021) 10:930. doi: 10.3390/cells10040930. PMID: 33920544 PMC8073519

[8] Fucikova J Moserova I Truxova I Hermanova I Vancurova I Partlova S . High hydrostatic pressure induces immunogenic cell death in human tumor cells. Int J Cancer. (2014) 135:1165–77. doi: 10.1002/ijc.28766. PMID: 24500981

[9] Vounckx M Tijtgat J Stevens L Dirven I Ilsen B Vandenbroucke F . A randomized phase II clinical trial of stereotactic body radiation therapy (SBRT) and systemic pembrolizumab with or without intratumoral avelumab/ipilimumab plus CD1c (BDCA-1)(+)/CD141 (BDCA-3)(+) myeloid dendritic cells in solid tumors. Cancer Immunol Immunother. (2024) 73:167. doi: 10.1007/s00262-024-03751-0. PMID: 38954010 PMC11219623

[10] Lamberti MJ Nigro A Mentucci FM Rumie Vittar NB Casolaro V Dal Col J . Dendritic cells and immunogenic cancer cell death: a combination for improving antitumor immunity. Pharmaceutics. (2020) 12:256. doi: 10.3390/pharmaceutics12030256. PMID: 32178288 PMC7151083

[11] Wang X Wang Y Zhang Y Shi H Liu K Wang F . Immune modulatory roles of radioimmunotherapy: biological principles and clinical prospects. Front Immunol. (2024) 15:1357101. doi: 10.3389/fimmu.2024.1357101. PMID: 38449871 PMC10915027

[12] Reits EA Hodge JW Herberts CA Groothuis TA Chakraborty M Wansley EK . Radiation modulates the peptide repertoire, enhances MHC class I expression, and induces successful antitumor immunotherapy. J Exp Med. (2006) 203:1259–71. doi: 10.1084/jem.20052494. PMID: 16636135 PMC3212727

[13] Lin AJ Roach M Bradley J Robinson C . Combining stereotactic body radiation therapy with immunotherapy: current data and future directions. Transl Lung Cancer Res. (2019) 8:107–15. doi: 10.21037/tlcr.2018.08.16. PMID: 30788240 PMC6351396

[14] Cheng SH Tu KY Lee HH . The dynamic duo: a narrative review on the synergy between stereotactic body radiotherapy and immunotherapy in lung cancer treatment (review). Oncol Rep. (2024) 52:96. doi: 10.3892/or.2024.8755. PMID: 38874014 PMC11188053

[15] Jiang J Li H Ma Q Liu J Ren F Song Y . Synergies between radiotherapy and immunotherapy: a systematic review from mechanism to clinical application. Front Immunol. (2025) 16:1554499. doi: 10.3389/fimmu.2025.1554499. PMID: 40861450 PMC12375553

[16] Vanpouille-Box C Alard A Aryankalayil MJ Sarfraz Y Diamond JM Schneider RJ . DNA exonuclease Trex1 regulates radiotherapy-induced tumour immunogenicity. Nat Commun. (2017) 8:15618. doi: 10.1038/ncomms15618. PMID: 28598415 PMC5472757

[17] Png S Tadepalli S Graves EE . Radiation-induced immune responses from the tumor microenvironment to systemic immunity. Cancers. (2025) 17:3849. doi: 10.3390/cancers17233849. PMID: 41375050 PMC12691506

[18] Deng L Liang H Xu M Yang X Burnette B Arina A . STING-dependent cytosolic DNA sensing promotes radiation-induced type I interferon-dependent antitumor immunity in immunogenic tumors. Immunity. (2014) 41:843–52. doi: 10.1016/j.immuni.2014.10.019. PMID: 25517616 PMC5155593

[19] Shang Z Wang L Zhou W . TREX1 exonuclease in immunity and disease. Int Immunol. (2025) 37:743–54. doi: 10.1093/intimm/dxaf037. PMID: 40627703

[20] Liu J Zhou J Wu M Hu C Yang J Li D . Low-dose total body irradiation can enhance systemic immune related response induced by hypo-fractionated radiation. Front Immunol. (2019) 10:317. doi: 10.3389/fimmu.2019.00317. PMID: 30873170 PMC6401363

[21] Fang L Ying S Xu X Wu D . TREX1 cytosolic DNA degradation correlates with autoimmune disease and cancer immunity. Clin Exp Immunol. (2023) 211:193–207. doi: 10.1093/cei/uxad017. PMID: 36745566 PMC10038326

[22] Guan H Zhang W Xie D Nie Y Chen S Sun X . Cytosolic release of mitochondrial DNA and associated cGAS signaling mediates radiation-induced hematopoietic injury of mice. Int J Mol Sci. (2023) 24:4020. doi: 10.3390/ijms24044020. PMID: 36835431 PMC9960871

[23] Du J Kageyama SI Yamashita R Tanaka K Okumura M Motegi A . Transposable elements potentiate radiotherapy-induced cellular immune reactions via RIG-I-mediated virus-sensing pathways. Commun Biol. (2023) 6:818. doi: 10.1038/s42003-023-05080-x. PMID: 37543704 PMC10404237

[24] Herrera FG Ronet C Ochoa de Olza M Barras D Crespo I Andreatta M . Low-dose radiotherapy reverses tumor immune desertification and resistance to immunotherapy. Cancer Discov. (2022) 12:108–33. doi: 10.1158/2159-8290.Cd-21-0003. PMID: 34479871 PMC9401506

[25] Guo Y Shen R Wang F Wang Y Xia P Wu R . Carbon ion irradiation induces DNA damage in melanoma and optimizes the tumor microenvironment based on the cGAS-STING pathway. J Cancer Res Clin Oncol. (2023) 149:6315–28. doi: 10.1007/s00432-023-04577-6. PMID: 36745223 PMC11798016

[26] Kim MS Kim W Park IH Kim HJ Lee E Jung JH . Radiobiological mechanisms of stereotactic body radiation therapy and stereotactic radiation surgery. Radiat Oncol J. (2015) 33:265–75. doi: 10.3857/roj.2015.33.4.265. PMID: 26756026 PMC4707209

[27] El Kaffas A Al-Mahrouki A Hashim A Law N Giles A Czarnota GJ . Role of acid sphingomyelinase and ceramide in mechano-acoustic enhancement of tumor radiation responses. J Natl Cancer Inst. (2018) 110:1009–18. doi: 10.1093/jnci/djy011. PMID: 29506145 PMC6136928

[28] Padilla F Brenner J Prada F Klibanov AL . Theranostics in the vasculature: bioeffects of ultrasound and microbubbles to induce vascular shutdown. Theranostics. (2023) 13:4079–101. doi: 10.7150/thno.70372. PMID: 37554276 PMC10405856

[29] Song CW Lee YJ Griffin RJ Park I Koonce NA Hui S . Indirect tumor cell death after high-dose hypofractionated irradiation: implications for stereotactic body radiation therapy and stereotactic radiation surgery. Int J Radiat Oncol Biol Phys. (2015) 93:166–72. doi: 10.1016/j.ijrobp.2015.05.016. PMID: 26279032 PMC4729457

[30] Takanen S Bottero M Nisticò P Sanguineti G . A systematic review on the impact of hypofractionated and stereotactic radiotherapy on immune cell subpopulations in cancer patients. Cancers (Basel). (2022) 14:5190. doi: 10.3390/cancers14215190. PMID: 36358608 PMC9653806

[31] Serre R Barlesi F Muracciole X Barbolosi D . Immunologically effective dose: a practical model for immuno-radiotherapy. Oncotarget. (2018) 9:31812–9. doi: 10.18632/oncotarget.25746. PMID: 30159124 PMC6112752

[32] Shen M Jiang X Peng Q Oyang L Ren Z Wang J . The cGAS–STING pathway in cancer immunity: mechanisms, challenges, and therapeutic implications. J Hematol Oncol. (2025) 18:40. doi: 10.1186/s13045-025-01691-5. PMID: 40188340 PMC11972543

[33] Chow J Hoffend NC Abrams SI Schwaab T Singh AK Muhitch JB . Radiation induces dynamic changes to the T cell repertoire in renal cell carcinoma patients. Proc Natl Acad Sci USA. (2020) 117:23721–9. doi: 10.1073/pnas.2001933117. PMID: 32900949 PMC7519245

[34] Dean I Lee CYC Tuong ZK Li Z Tibbitt CA Willis C . Rapid functional impairment of natural killer cells following tumor entry limits anti-tumor immunity. Nat Commun. (2024) 15:683. doi: 10.1038/s41467-024-44789-z. PMID: 38267402 PMC10808449

[35] Qiu Y Ke S Chen J Qin Z Zhang W Yuan Y . FOXP3+ regulatory T cells and the immune escape in solid tumours. Front Immunol. (2022) 13:982986. doi: 10.3389/fimmu.2022.982986. PMID: 36569832 PMC9774953

[36] Zhao C Tang Q Yang C Zhou L Peng J Zhang T . Stereotactic body radiation therapy suppresses myeloid-derived suppressor cells by regulating miR-21/Sorbin and SH3 domain-containing protein 1 axis. Hum Exp Toxicol. (2024) 43:9603271241261307. doi: 10.1177/09603271241261307. PMID: 38874389

[37] Ostrand-Rosenberg S Horn LA Ciavattone NG . Radiotherapy both promotes and inhibits myeloid-derived suppressor cell function: novel strategies for preventing the tumor-protective effects of radiotherapy. Front Oncol. (2019) 9:215. doi: 10.3389/fonc.2019.00215. PMID: 31001479 PMC6454107

[38] Bergerud KMB Berkseth M Pardoll DM Ganguly S Kleinberg LR Lawrence J . Radiation therapy and myeloid-derived suppressor cells: breaking down their cancerous partnership. Int J Radiat Oncol Biol Phys. (2024) 119:42–55. doi: 10.1016/j.ijrobp.2023.11.050. PMID: 38042450 PMC11082936

[39] Haist M Stege H Grabbe S Bros M . The functional crosstalk between myeloid-derived suppressor cells and regulatory T cells within the immunosuppressive tumor microenvironment. Cancers (Basel). (2021) 13:210. doi: 10.3390/cancers13020210. PMID: 33430105 PMC7827203

[40] Bullock K Richmond A . Suppressing MDSC recruitment to the tumor microenvironment by antagonizing CXCR2 to enhance the efficacy of immunotherapy. Cancers (Basel). (2021) 13:6293. doi: 10.3390/cancers13246293. PMID: 34944914 PMC8699249

[41] Xu Y Ying K . Research progress on neutrophil extracellular traps in tumor. Zhejiang Da Xue Xue Bao Yi Xue Ban. (2020) 49:107–12. doi: 10.3785/j.issn.1008-9292.2020.02.11. PMID: 32621421 PMC8800663

[42] Shinde-Jadhav S Mansure JJ Rayes RF Marcq G Ayoub M Skowronski R . Role of neutrophil extracellular traps in radiation resistance of invasive bladder cancer. Nat Commun. (2021) 12:2776. doi: 10.1038/s41467-021-23086-z. PMID: 33986291 PMC8119713

[43] Webster M Podgorsak A Li F Zhou Y Jung H Yoon J . New approaches in radiotherapy. Cancers (Basel). (2025) 17:1980. doi: 10.3390/cancers17121980. PMID: 40563630 PMC12190917

[44] Klug F Prakash H Huber PE Seibel T Bender N Halama N . Low-dose irradiation programs macrophage differentiation to an iNOS^+^/M1 phenotype that orchestrates effective T cell immunotherapy. Cancer Cell. (2013) 24:589–602. doi: 10.1016/j.ccr.2013.09.014. PMID: 24209604

[45] Kalbasi A Komar C Tooker GM Liu M Lee JW Gladney WL . Tumor-derived CCL2 mediates resistance to radiotherapy in pancreatic ductal adenocarcinoma. Clin Cancer Res. (2017) 23:137–48. doi: 10.1158/1078-0432.Ccr-16-0870. PMID: 27354473 PMC5195913

[46] Darragh LB Gadwa J Pham TT Van Court B Neupert B Olimpo NA . Elective nodal irradiation mitigates local and systemic immunity generated by combination radiation and immunotherapy in head and neck tumors. Nat Commun. (2022) 13:7015. doi: 10.1038/s41467-022-34676-w. PMID: 36385142 PMC9668826

[47] Zhou X Zhou L Yao Z Huang M Gong Y Zou B . Safety and tolerability of low-dose radiation and stereotactic body radiotherapy + sintilimab for treatment-naïve stage IV PD-L1+ non-small cell lung cancer patients. Clin Cancer Res. (2023) 29:4098–108. doi: 10.1158/1078-0432.Ccr-23-0315. PMID: 37581611

[48] Novikov SN Krzhivitskii PI Melnik YS Valitova AA Bryantseva ZV Akulova IA . Atlas of sentinel lymph nodes in early breast cancer using single-photon emission computed tomography: implication for lymphatic contouring. Radiat Oncol J. (2021) 39:8–14. doi: 10.3857/roj.2020.00871. PMID: 33794569 PMC8024181

[49] Ganswindt U Schilling D Müller AC Bares R Bartenstein P Belka C . Distribution of prostate sentinel nodes: a SPECT-derived anatomic atlas. Int J Radiat Oncol Biol Phys. (2011) 79:1364–72. doi: 10.1016/j.ijrobp.2010.01.012. PMID: 20797823

[50] Mandal K Barik GK Santra MK . Overcoming resistance to anti-PD-L1 immunotherapy: mechanisms, combination strategies, and future directions. Mol Cancer. (2025) 24:246. doi: 10.1186/s12943-025-02400-z. PMID: 41057853 PMC12505684

[51] Passelli K Repáraz D Herrera FG . Chapter five - opportunities and challenges of low-dose radiation to enable immunotherapy efficacy. In: Mirjolet C Galluzzi L , editors. International Review of Cell and Molecular Biology, vol. 378 . Academic Press (2023). p. 137–56. 10.1016/bs.ircmb.2023.03.010 37438016

[52] Theelen W Peulen HMU Lalezari F Van Der Noort V De Vries JF Aerts J . Effect of pembrolizumab after stereotactic body radiotherapy vs pembrolizumab alone on tumor response in patients with advanced non-small cell lung cancer: Results of the PEMBRO-RT phase 2 randomized clinical trial. JAMA Oncol. (2019) 5:1276–82. doi: 10.1001/jamaoncol.2019.1478. PMID: 31294749 PMC6624814

[53] Welsh J Menon H Chen D Verma V Tang C Altan M . Pembrolizumab with or without radiation therapy for metastatic non-small cell lung cancer: a randomized phase I/II trial. J Immunother Cancer. (2020) 8:001001. doi: 10.1136/jitc-2020-001001. PMID: 33051340 PMC7555111

[54] Qu FJ Zhou Y Wu S . Progress of immune checkpoint inhibitors therapy for non-small cell lung cancer with liver metastases. Br J Cancer. (2024) 130:165–75. doi: 10.1038/s41416-023-02482-w. PMID: 37945751 PMC10803805

[55] Horst AK Neumann K Diehl L Tiegs G . Modulation of liver tolerance by conventional and nonconventional antigen-presenting cells and regulatory immune cells. Cell Mol Immunol. (2016) 13:277–92. doi: 10.1038/cmi.2015.112. PMID: 27041638 PMC4856800

[56] Shi S Ou X Liu C Wen H Ke J . Research progress of HIF-1a on immunotherapy outcomes in immune vascular microenvironment. Front Immunol. (2025) 16:1549276. doi: 10.3389/fimmu.2025.1549276. PMID: 39981236 PMC11839635

[57] Chen HHW Kuo MT . Improving radiotherapy in cancer treatment: promises and challenges. Oncotarget. (2017) 8:62742–58. doi: 10.18632/oncotarget.18409. PMID: 28977985 PMC5617545

[58] Dai Y Tian X Ye X Gong Y Xu L Jiao L . Role of the TME in immune checkpoint blockade resistance of non-small cell lung cancer. Cancer Drug Resist. (2024) 7:52. doi: 10.20517/cdr.2024.166. PMID: 39802954 PMC11724356

[59] De Visser KE Joyce JA . The evolving tumor microenvironment: from cancer initiation to metastatic outgrowth. Cancer Cell. (2023) 41:374–403. doi: 10.1016/j.ccell.2023.02.016. PMID: 36917948

[60] Sankar K Pearson AN Worlikar T Perricone MD Holcomb EA Mendiratta-Lala M . Impact of immune tolerance mechanisms on the efficacy of immunotherapy in primary and secondary liver cancers. Transl Gastroenterol Hepatol. (2023) 8:29. doi: 10.21037/tgh-23-11. PMID: 37601739 PMC10432235

[61] Zhao J Kuang D Cheng X Geng J Huang Y Zhao H . Molecular mechanism of colorectal cancer and screening of molecular markers based on bioinformatics analysis. Open Life Sci. (2023) 18:20220687. doi: 10.1515/biol-2022-0687. PMID: 37954103 PMC10638842

[62] Luan L Dai Y Shen T Yang C Chen Z Liu S . Development of a novel hypoxia-immune-related LncRNA risk signature for predicting the prognosis and immunotherapy response of colorectal cancer. Front Immunol. (2022) 13:951455. doi: 10.3389/fimmu.2022.951455. PMID: 36189298 PMC9516397

[63] Ai X Cai Y Chu Q Han C Lu Y Qin S . Combination of radiation therapy and immunotherapy for non-small cell lung cancer: peer exchange on frontier academic topics. Zhongguo Fei Ai Za Zhi. (2020) 23:532–40. doi: 10.3779/j.issn.1009-3419.2020.102.24. PMID: 32517461 PMC7309548

[64] Zhang Z Liu X Chen D Yu J . Radiotherapy combined with immunotherapy: the dawn of cancer treatment. Signal Transd Targ Ther. (2022) 7:258. doi: 10.1038/s41392-022-01102-y. PMID: 35906199 PMC9338328

[65] Qian X Fang Z Jiang W Chou J Lu Y Jabbour SK . The optimal stereotactic body radiotherapy dose with immunotherapy for pulmonary oligometastases: a retrospective cohort study. J Thorac Dis. (2024) 16:7072–85. doi: 10.21037/jtd-24-1624. PMID: 39552865 PMC11565358

[66] Mekers VE Span PN Looman M Van Den Bogaard L Kho V Melchers WJG . The radiotherapy induced cyclic GMP-AMP-synthase re-localization and immune signature predicts HNSCC treatment efficacy. Commun Med. (2026) 6:75. doi: 10.1038/s43856-025-01336-1. PMID: 41545483 PMC12873430

[67] Huang J Theelen W Belcaid Z Najjar M Van Der Geest D Singh D . Combination of pembrolizumab and radiotherapy induces systemic antitumor immune responses in immunologically cold non-small cell lung cancer. Nat Cancer. (2025) 6:1676–92. doi: 10.1038/s43018-025-01018-w. PMID: 40696153 PMC12559004

[68] McCall NS McGinnis HS Janopaul-Naylor JR Kesarwala AH Tian S Stokes WA . Impact of radiation dose to the immune cells in unresectable or stage III non-small cell lung cancer in the durvalumab era. Radiother Oncol. (2022) 174:133–40. doi: 10.1016/j.radonc.2022.07.015. PMID: 35870727

[69] Eckstein J Gogineni E Sidiqi B Lisser N Parashar B . Effect of immunotherapy and stereotactic body radiation therapy sequencing on local control and survival in patients with spine metastases. Adv Radiat Oncol. (2023) 8:101179. doi: 10.1016/j.adro.2023.101179. PMID: 36896213 PMC9991541

[70] Demaria S Coleman CN Formenti SC . Radiotherapy: changing the game in immunotherapy. Trends Cancer. (2016) 2:286–94. doi: 10.1016/j.trecan.2016.05.002. PMID: 27774519 PMC5070800

[71] Woody S Hegde A Arastu H Peach MS Sharma N Walker P . Survival is worse in patients completing immunotherapy prior to SBRT/SRS compared to those receiving it concurrently or after. Front Oncol. (2022) 12:785350. doi: 10.3389/fonc.2022.785350. PMID: 35692764 PMC9184512

[72] Peng H Moore C Saha D Jiang S Timmerman R . Understanding the PULSAR effect in combined radiotherapy and immunotherapy using transformer-based attention mechanisms. Front Oncol. (2024) 14:1497351. doi: 10.3389/fonc.2024.1497351. PMID: 39687891 PMC11647037

[73] Peng H Deng J Jiang S Timmerman R . Rethinking the potential role of dose painting in personalized ultra-fractionated stereotactic adaptive radiotherapy. Front Oncol. (2024) 14:1357790. doi: 10.3389/fonc.2024.1357790. PMID: 38571510 PMC10987838

[74] Skorupan N Palestino Dominguez M Ricci SL Alewine C . Clinical strategies targeting the tumor microenvironment of pancreatic ductal adenocarcinoma. Cancers (Basel). (2022) 14:4209. doi: 10.3390/cancers14174209. PMID: 36077755 PMC9454553

[75] Mills BN Qiu H Drage MG Chen C Mathew JS Garrett-Larsen J . Modulation of the human pancreatic ductal adenocarcinoma immune microenvironment by stereotactic body radiotherapy. Clin Cancer Res. (2022) 28:150–62. doi: 10.1158/1078-0432.Ccr-21-2495. PMID: 34862242 PMC8738140

[76] Allard B Turcotte M Stagg J . CD73-generated adenosine: orchestrating the tumor-stroma interplay to promote cancer growth. J BioMed Biotechnol. (2012) 2012:485156. doi: 10.1155/2012/485156. PMID: 23125525 PMC3482007

[77] Ye J Gavras NW Keeley DC Hughson AL Hannon G Vrooman TG . CD73 and PD-L1 dual blockade amplifies antitumor efficacy of SBRT in murine PDAC models. J Immunother Cancer. (2023) 11:006842. doi: 10.1136/jitc-2023-006842. PMID: 37142292 PMC10163599

[78] Wang J Chang CY Yang X Zhou F Liu J Feng Z . Leukemia inhibitory factor, a double-edged sword with therapeutic implications in human diseases. Mol Ther. (2023) 31:331–43. doi: 10.1016/j.ymthe.2022.12.016. PMID: 36575793 PMC9931620

[79] Ye J Qin SS Hughson AL Hannon G Salama NA Vrooman TG . Blocking LIF and PD-L1 enhances the antitumor efficacy of SBRT in murine PDAC models. J Immunother Cancer. (2025) 13:010820. doi: 10.1136/jitc-2024-010820. PMID: 40341024 PMC12067785

[80] Qin VM Haynes NM D'Souza C Neeson PJ Zhu JJ . CAR-T plus radiotherapy: a promising combination for immunosuppressive tumors. Front Immunol. (2021) 12:813832. doi: 10.3389/fimmu.2021.813832. PMID: 35095911 PMC8790144

[81] Manjunath SH Cohen AD Lacey SF Davis MM Garfall AL Melenhorst JJ . The safety of bridging radiation with anti-BCMA CAR T-cell therapy for multiple myeloma. Clin Cancer Res. (2021) 27:6580–90. doi: 10.1158/1078-0432.Ccr-21-0308. PMID: 34526365 PMC8639780

[82] Omolekan TO Folahan JT Tesfay MZ Mohan H Dutta O Rahimian L . Viral warfare: unleashing engineered oncolytic viruses to outsmart cancer’s defenses. Front Immunol. (2025) 16:1618751. doi: 10.3389/fimmu.2025.1618751. PMID: 40927720 PMC12414947

[83] Waxman ES Lee Gerber D . Pseudoprogression and immunotherapy phenomena. J Adv Pract Oncol. (2020) 11:723–31. doi: 10.6004/jadpro.2020.11.7.6. PMID: 33575068 PMC7646636

[84] Aoki H Shichino S Matsushima K Ueha S . Revealing clonal responses of tumor-reactive T-cells through T cell receptor repertoire analysis. Front Immunol. (2022) 13:807696. doi: 10.3389/fimmu.2022.807696. PMID: 35154125 PMC8829044

[85] Boonstra PA Wind TT Van Kruchten M Schuuring E Hospers GAP Van Der Wekken AJ . Clinical utility of circulating tumor DNA as a response and follow-up marker in cancer therapy. Cancer Metastas Rev. (2020) 39:999–1013. doi: 10.1007/s10555-020-09876-9. PMID: 32367253 PMC7497299

[86] Boniface CT Spellman PT . Blood, toil, and taxoteres: biological determinants of treatment-induced ctDNA dynamics for interpreting tumor response. Pathol Oncol Res. (2022) 28:1610103. doi: 10.3389/pore.2022.1610103. PMID: 35665409 PMC9160182

[87] Frank MS Andersen CSA Ahlborn LB Pallisgaard N Bodtger U Gehl J . Circulating tumor DNA monitoring reveals molecular progression before radiologic progression in a real-life cohort of patients with advanced non-small cell lung cancer. Cancer Res Commun. (2022) 2:1174–87. doi: 10.1158/2767-9764.Crc-22-0258. PMID: 36969747 PMC10035379

[88] Adhit KK Wanjari A Menon S K S . Liquid biopsy: an evolving paradigm for non-invasive disease diagnosis and monitoring in medicine. Cureus. (2023) 15:e50176. doi: 10.7759/cureus.50176. PMID: 38192931 PMC10772356

[89] Zhang H Dohopolski M Stojadinovic S Schmitt LG Anand S Kim H . Multiomics-based outcome prediction in personalized ultra-fractionated stereotactic adaptive radiotherapy (PULSAR). Cancers (Basel). (2024) 16:3425. doi: 10.3390/cancers16193425. PMID: 39410044 PMC11475788

[90] Peng L Bin Y Ding P Chen L Zeng H Xu Z . Integrated circulating tumor DNA and T cell repertoire predict radiotherapeutic response and outcome in non-small cell lung cancer patients with brain metastasis. Cancer Commun (Lond). (2023) 43:625–9. doi: 10.1002/cac2.12410. PMID: 36815673 PMC10174081

[91] Sánchez-Herrero E Serna-Blasco R Robado De Lope L González-Rumayor V Romero A Provencio M . Circulating tumor DNA as a cancer biomarker: an overview of biological features and factors that may impact on ctDNA analysis. Front Oncol. (2022) 12:943253. doi: 10.3389/fonc.2022.943253. PMID: 35936733 PMC9350013

[92] MacManus M Kirby L Blyth B Banks O Martin OA Yeung MM . Early circulating tumor DNA dynamics at the commencement of curative-intent radiotherapy or chemoradiotherapy for NSCLC. Clin Transl Radiat Oncol. (2023) 43:100682. doi: 10.1016/j.ctro.2023.100682. PMID: 37808452 PMC10551836

[93] Thompson JC Scholes DG Carpenter EL Aggarwal C . Molecular response assessment using circulating tumor DNA (ctDNA) in advanced solid tumors. Br J Cancer. (2023) 129:1893–902. doi: 10.1038/s41416-023-02445-1. PMID: 37789101 PMC10703899

